# Platelet and Haemostasis are the Main Targets in Severe Cases of COVID-19 Infection; a System Biology Study 

**DOI:** 10.22037/aaem.v9i1.1108

**Published:** 2021-03-14

**Authors:** Mona Zamanian-Azodi, Babak Arjmand, Mohammadreza Razzaghi, Mostafa Rezaei Tavirani, Alireza Ahmadzadeh, Mohammad Rostaminejad

**Affiliations:** 1 Proteomics Research Center, Faculty of Paramedical Sciences, Shahid Beheshti University of Medical Sciences, Tehran, Iran.; 2 Cell Therapy and Regenerative Medicine Research Center, Endocrinology and Metabolism Molecular-Cellular Sciences Institute, Tehran University of Medical Sciences, Tehran, Iran.; 3 Laser Application in Medical Sciences Research Center, Shahid Beheshti University of Medical Sciences, Tehran, Iran.; 4 Gastroenterology and Liver Diseases Research Center, Research Institute for Gastroenterology and Liver Diseases, Shahid Beheshti University of Medical Sciences, Tehran, Iran.

**Keywords:** COVID-19, Proteins, Bioinformatics, Computational Biology, Network analysis

## Abstract

**Introduction:**

Many proteomics-based and bioinformatics-based efforts are made to detect the molecular mechanism of COVID-19 infection. Identification of the main protein targets and pathways of severe cases of COVID-19 infection is the aim of this study.

**Methods:**

Published differentially expressed proteins were screened and the significant proteins were investigated via protein-protein interaction network using Cytoscape software V. 3.7.2 and STRING database. The studied proteins were assessed via action map analysis to determine the relationship between individual proteins using CluePedia. The related biological terms were investigated using ClueGO and the terms were clustered and discussed.

**Results:**

Among the 35 queried proteins, six of them (FGA, FGB, FGG, and FGl1 plus TLN1 and THBS1) were identified as critical proteins. A total of 38 biological terms, clustered in 4 groups, were introduced as the affected terms. “Platelet degranulation” and “hereditary factor I deficiency disease” were introduced as the main class of the terms disturbed by COVID-19 virus.

**Conclusion:**

It can be concluded that platelet damage and disturbed haemostasis could be the main targets in severe cases of coronavirus infection. It is vital to follow patients’ condition by examining the introduced critical differentially expressed proteins (DEPs).

## Introduction

COVID-19 infection resulted in difficulties all over the world and for all the different races of human beings in all countries. In addition, it has imposed complex effects on patients’ lifestyle, which lead to manifestation of other conditions such as diabetes, cancers, and other types of disorders, and has thus attracted the attention of researchers and they want to solve this problem (1-3). Since understanding the molecular mechanism of the diseases is fundamental in diagnosis and therapy of diseases, many efforts are made to study the molecular aspect of COVID-19 infection (4-6). 

Proteomics and informatics are two suitable methods for finding the molecular mechanism of different kinds of diseases (7, 8). Since proteomics is a high-throughput method, results of proteomics are reliable data that can be interpreted and analyzed via informatics (9, 10). Network analysis based on graph theory is a method in bioinformatics, which is widely applied for evaluating diseases in medical sciences (11, 12). Differentially expressed proteins (DEPs) bind to the other proteins based on affinity, and form a network of nodes, which are linked by edges (13). The constructed network contains useful information about the elements of the network (14). Action map is another useful method for determining the relationship between the queried DEPs. Possible inhibition, activation, reaction, binding, and regulation roles of a protein related to the neighbors can be identified via action map analysis (15).

Gene ontology is another molecular analysis that can be used to detect the pathways and biological processes that are related to the studied proteins. Many diseases are assessed via gene ontology method to find the critical dysregulated pathways and biological processes (16, 17). 

In the present study, DEPs of severe cases of COVID-19 are extracted from a paper by Ting Shu et al. and are investigated via network analysis, action map assessment, and gene ontology examination. In the report of Ting Shu et al., plasma protein expression changes of patients in the cases of fatal, severe, and mild conditions are compared with the controls. Here, the severe cases of COVID-19 were selected to be assessed and their significant DEPs were investigated.

## Methods

In this bioinformatics study, 35 differentially expressed proteins based on fold change ≥ 1.5 and p-value ≤ 0.01, which were identified by evaluating protein expressions in severe cases of COVID-19 versus healthy people, were extracted from the paper published by Ting Shu et al. (18). The differentially expressed proteins were included in an interactome unit using “protein query” of STRING database via Cytoscape software 3.7.2. The network including a main connected component and two isolated proteins was constructed. Furthermore, to understand the type of interactions between the nodes, action map analysis was investigated. For this purpose, activation, inhibition, binding, and regulation actions were evaluated using CluePedia v1.5.7. The biological terms related to the 35 DEPs were investigated using ClueGO 2.5.7 from REACTOME_Pathways_08.05.2020, CLINVAR_Human-diseases_08.05.2020, KEGG_08.05.2020, and WikiPathways_08.05.2020.

In the statistical analysis, protein expression values were determined based on mean value of data. Kapa scoring was set to 0.4. Additionally, Term P value corrected with Bonferroni step down, group P value, and group P value corrected with Bonferroni step down were ≤ 0.01 in gene ontology analysis. The protocol of study was approved by Ethics Committee of Shahid Beheshti University of Medical Sciences, Tehran, Iran (Ethics code: IR.SBMU.RETECH.REC.1399.355).

## Results

**Figure 1:**
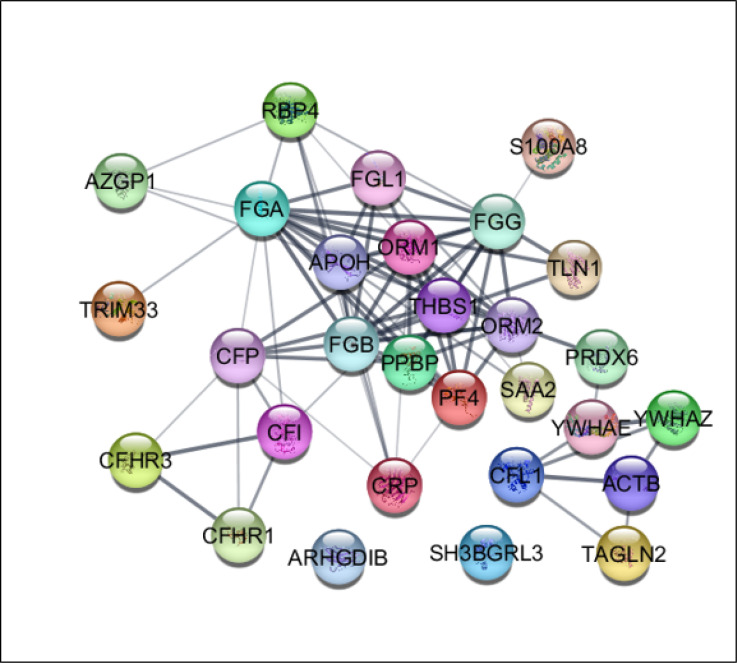
The queried 35 differentially expressed proteins (DEPs) are included in a network using STRING database and Cytoscape software

**Figure 2:**
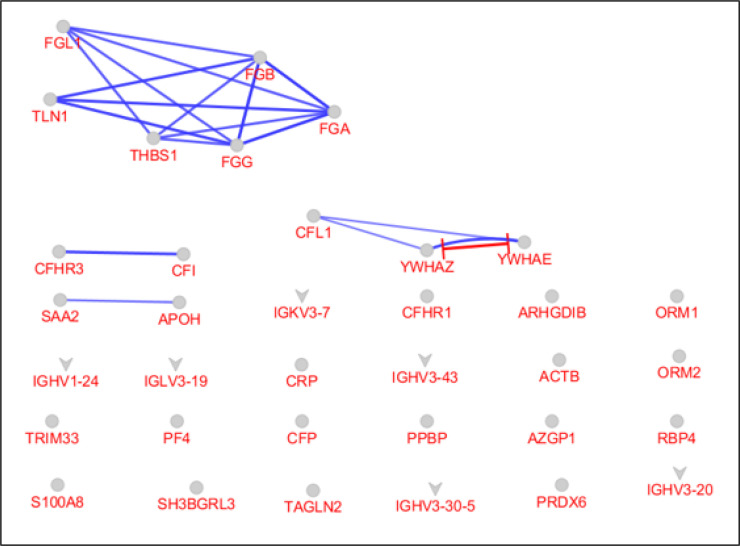
The action map for the 35 queried differentially expressed proteins (DEPs) via CluePedia. The blue and red colors of edges refer to binding and inhibition actions

Except for ARHGDIB and SH3BGRL3 the other DEPs were included in the network by undirected edges. As shown in figure 1, a compacted region, which is mainly formed by various types of fibrinogen chains, has appeared as a central part of the constructed network. This finding is confirmed by action map (see figure 2). FGA, FGB, FGG, and FGl1 plus TLN1 and THBS1 are connected together in action map. YWHAZ, YWHAE, and CFL1 that are located in figure 1, formed a triple unit in the action map. Since the network is not a scale free type, centrality analysis was not applied to find the central nodes such as hubs or bottlenecks.

**Figure 3:**
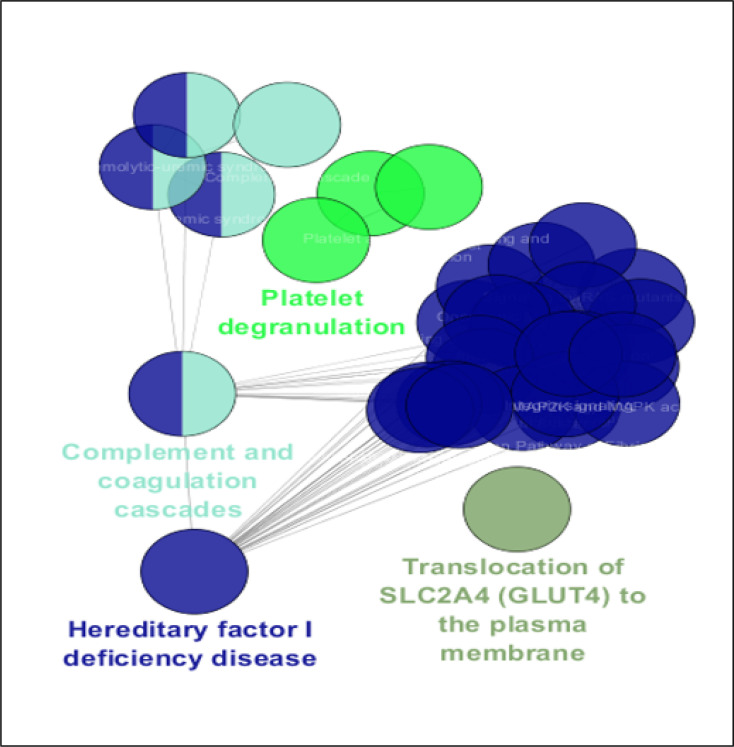
Gene ontology results related to the 35 queried differentially expressed proteins (DEPs). The 38 terms are classified in the four groups

**Figure 4:**
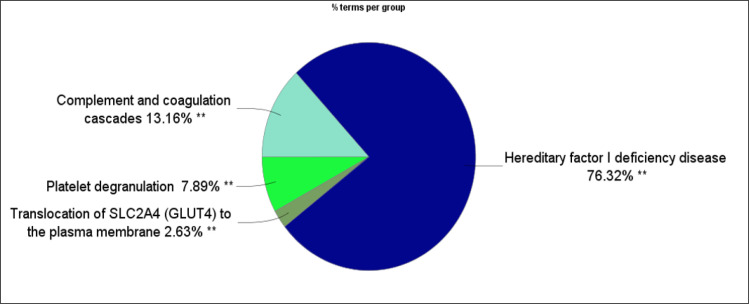
Frequency of four classes of biological terms as a pie chart. Different colors indicate designated groups of terms

A total of 38 biological terms related to the 35 DEPs are shown in figure 3 and table 1. The terms are grouped in four classes. The smallest group includes only one term (Translocation of SLC2A4 (GLUT4) to the plasma membrane), while Hereditary factor I deficiency disease, as the largest group, includes 29 terms. Frequency of groups of terms is represented in figure 4.

## Discussion

Efforts of researchers to solve COVID-19 infection problems led to production of large numbers of publications. Proteomics and bioinformatics are two powerful methods that have been frequently applied in molecular studies of COVID-19 (19, 20). In the present study, bioinformatics evaluation of plasma proteome of patients with severe COVID-19 revealed a new perspective of the disease. As shown in figure 1, a total of 35 significant DEPs are connected as an interactome unit to create a new concept about COVID-19 pandemic. Apart from two proteins, the other DEPs are interacted in a heterogeneous way and several nodes form a compact area as a cluster. This compact zone is shown as a cluster including six proteins in figure 2. It seems that these six proteins (including four varieties of fibrinogen, talin-1 (TLN1), and thrombospondin-1 (THBS1)) play a critical role among the 35 queried DEPs in response to the COVID-19 infection. Investigation indicates that regulation of talin-1 effects platelet activation (21). The role of thrombospondin-1 in stimulating platelet aggregation is reported by Jeff S. Isenberg et al. (22).

The biological terms that are connected to the DEPs are shown in figure 3 and table 1. To find the terms that are connected to the six critical DEPs, the terms that were related to at least one node of these critical proteins were determined. Findings indicate that among the 31 terms in cluster 4, there are 29 terms (about 94%) that are linked to the several members of the six DEPs. All terms of cluster 2 are linked to the members of the critical DEPs, while the single term of cluster 1 has no connection to the critical set of DEPs. Four terms (80%) of cluster 3 members have no connection to the mentioned DEPs. Based on the analysis, it can be concluded that, clusters 2 and 4 (“platelet degranulation” and “hereditary factor I deficiency disease”, respectively) are the prominent terms that are dysregulated in response to COVID-19 infection.

Hereditary factor I deficiency disease or fibrinogen deficiency is a blood disorder that is accompanied with decreased level of fibrinogen (afibrinogenemia, hypofibrinogenemia) or disturbed quality of fibrinogen (dysfibrinogenemia) in circulation (23). As previously known, fibrinogen has a noticeable role in normal haemostasis in human body. It is the key element of promotion of fibrinolysis, clot formation, and platelet aggregation processes (24). As depicted in figure 4, about 76% of the determined biological terms are related to the “Hereditary factor I deficiency disease”.

The second cluster of terms is “platelet degranulation” class of pathway, which includes 3 terms. Participation in haemostasis is the well-known role of platelet in blood. An essential process in response to vascular damage is platelet adhesion, which leads to initiation of thrombus creation at the time of hemorrhage and promotes wound healing (25, 26). There is a similar function that the two biological terms (“Hereditary factor I deficiency disease” and “platelet degranulation”) are involved in: haemostasis. It can be concluded that disturbed haemostasis is the main dysfunction in severe cases of COVID-19. What is more, clinical features of infection with coronavirus (1, 27) support the findings of our study.

Since COVID-19 is a new disease, more data and sufficient patients are required to analyze the molecular events related to the promotion of infection. Complementary investigations regarding different parameters such as age, geography, race, and other conditions are recommended to achieve a better understanding of the molecular mechanism of COVID-19.

**Table 1:** List of 38 biological terms related to the queried differentially expressed proteins (DEPs)

**GO Term**	**N**	**AGs %**	**Associated Genes Found**
Translocation of SLC2A4 (GLUT4) to the plasma membrane	1	4.17	[ACTB, YWHAE, YWHAZ]
Platelet degranulation	2	9.30	[APOH, CFL1, FGA, FGB, FGG, ORM1, ORM2, PF4, PPBP, TAGLN2, THBS1, TLN1]
Platelet activation, signaling and aggregation	2	4.94	[APOH, CFL1, FGA, FGB, FGG, ORM1, ORM2, PF4, PPBP, TAGLN2, THBS1, TLN1, YWHAZ]
Response to elevated platelet cytosolic Ca2+	2	8.96	[APOH, CFL1, FGA, FGB, FGG, ORM1, ORM2, PF4, PPBP, TAGLN2, THBS1, TLN1]
Hemolytic-uremic syndrome	3	33.33	[CFHR1, CFHR3, CFI]
Atypical hemolytic uremic syndrome	3	33.33	[CFHR1, CFHR3, CFI]
Complement and coagulation cascades	3	7.06	[CFHR1, CFHR3, CFI, FGA, FGB, FGG]
Complement cascade	3	8.62	[CFHR1, CFHR3, CFI, CFP, CRP]
Regulation of Complement cascade	3	8.51	[CFHR1, CFHR3, CFI, CFP]
Hemolytic-uremic syndrome	4	33.33	[CFHR1, CFHR3, CFI]
Hereditary factor I deficiency disease	4	100.00	[CFI, FGA, FGB, FGG]
Dysfibrinogenemia, congenital	4	100.00	[FGA, FGB, FGG]
Afibrinogenemia, congenital	4	100.00	[FGA, FGB, FGG]
Atypical hemolytic uremic syndrome	4	33.33	[CFHR1, CFHR3, CFI]
Complement and coagulation cascades	4	7.06	[CFHR1, CFHR3, CFI, FGA, FGB, FGG]
Platelet activation	4	4.03	[ACTB, FGA, FGB, FGG, TLN1]
Common Pathway of Fibrin Clot Formation	4	18.18	[FGA, FGB, FGG, PF4]
Formation of Fibrin Clot (Clotting Cascade)	4	10.26	[FGA, FGB, FGG, PF4]
Integrin cell surface interactions	4	4.71	[FGA, FGB, FGG, THBS1]
Integrin signaling	4	14.81	[FGA, FGB, FGG, TLN1]
GRB2:SOS provides linkage to MAPK signaling for Integrins	4	26.67	[FGA, FGB, FGG, TLN1]
p130Cas linkage to MAPK signaling for integrins	4	26.67	[FGA, FGB, FGG, TLN1]
MAP2K and MAPK activation	4	12.50	[ACTB, FGA, FGB, FGG, TLN1]
Regulation of TLR by endogenous ligand	4	21.05	[FGA, FGB, FGG, S100A8]
Signaling by moderate kinase activity BRAF mutants	4	10.64	[ACTB, FGA, FGB, FGG, TLN1]
Signaling by high-kinase activity BRAF mutants	4	13.89	[ACTB, FGA, FGB, FGG, TLN1]
Signaling by RAS mutants	4	10.64	[ACTB, FGA, FGB, FGG, TLN1]
Signaling by BRAF and RAF fusions	4	7.46	[ACTB, FGA, FGB, FGG, TLN1]
Paradoxical activation of RAF signaling by kinase inactive BRAF	4	10.64	[ACTB, FGA, FGB, FGG, TLN1]
Oncogenic MAPK signaling	4	6.76	[ACTB, FGA, FGB, FGG, TLN1]
Platelet Aggregation (Plug Formation)	4	10.26	[FGA, FGB, FGG, TLN1]
Signaling downstream of RAS mutants	4	10.64	[ACTB, FGA, FGB, FGG, TLN1]
Regulation of Complement cascade	4	8.51	[CFHR1, CFHR3, CFI, CFP]
Selenium Micronutrient Network	4	5.43	[CRP, FGA, FGB, FGG, SAA2]
Folate Metabolism	4	6.85	[CRP, FGA, FGB, FGG, SAA2]
Blood Clotting Cascade	4	13.04	[FGA, FGB, FGG]
Human Complement System	4	7.07	[CFI, CFP, CRP, FGA, FGB, FGG, THBS1]
Fibrin Complement Receptor 3 Signaling Pathway	4	7.14	[FGA, FGB, FGG]

The terms are extracted from Ontology Source; REACTOME_Pathways_08.05.2020, CLINVAR_Human-diseases_08.05.2020, KEGG_08.05.2020, WikiPathways_08.05.2020. Term P Value, term P Value Corrected with Bonferroni step down, group P Value, and term P Value Corrected with Bonferroni step down ≤ 0.01 were considered. GO: gene ontology; N: number of group; AGs: associated genes.

## Conclusion:

It can be concluded that platelet damage and disturbed haemostasis could be the main targets in severe cases of coronavirus infection. It is vital to follow patients’ condition by examining the introduced critical DEPs. 

## Conflict of interest

There is no conflict of interest.
